# Teledermatology: Comparison of Store-and-Forward Versus Live Interactive Video Conferencing

**DOI:** 10.2196/11871

**Published:** 2018-10-24

**Authors:** Titus Josef Brinker, Achim Hekler, Christof von Kalle, Dirk Schadendorf, Stefan Esser, Carola Berking, Martina T Zacher, Wiebke Sondermann, Niels Grabe, Theresa Steeb, Jochen Sven Utikal, Lars E French, Alexander H Enk

**Affiliations:** 1 Department of Translational Oncology National Center for Tumor Diseases German Cancer Research Center Heidelberg Germany; 2 Department of Dermatology University Hospital Heidelberg University of Heidelberg Heidelberg Germany; 3 German Cancer Consortium University of Heidelberg Heidelberg Germany; 4 Department of Dermatology University Hospital Essen University of Duisburg-Essen Essen Germany; 5 University Hospital Zurich University of Zurich Zurich Switzerland; 6 Department of Dermatology and Allergology University Medical Center Munich Ludwig Maximilian University of Munich Munich Germany; 7 BioQuant Hamamatsu Tissue Imaging and Analysis Center (TIGA) University of Heidelberg Heidelberg Germany; 8 German Cancer Research Center Skin Cancer Unit University of Heidelberg Mannheim Germany; 9 Department of Dermatology University Hospital of Zurich University of Zurich Zurich Switzerland

**Keywords:** teledermatology, live video conferencing, store-and-forward teledermatology, mobile phone, wait time, live interactive

## Abstract

A decreasing number of dermatologists and an increasing number of patients in Western countries have led to a relative lack of clinicians providing expert dermatologic care. This, in turn, has prolonged wait times for patients to be examined, putting them at risk. Store-and-forward teledermatology improves patient access to dermatologists through asynchronous consultations, reducing wait times to obtain a consultation. However, live video conferencing as a synchronous service is also frequently used by practitioners because it allows immediate interaction between patient and physician. This raises the question of which of the two approaches is superior in terms of quality of care and convenience. There are pros and cons for each in terms of technical requirements and features. This viewpoint compares the two techniques based on a literature review and a clinical perspective to help dermatologists assess the value of teledermatology and determine which techniques would be valuable in their practice.

## Introduction

### Background

Teledermatology, originating in 1995, was one of the first telemedicine services to be implemented, with other medical specialties following later [1-3]. Aging populations and a relative lack of dermatologists have prolonged wait times in the Western world, increasing the demand for new, more efficient strategies to render dermatologic care [4]. Mobile phones have overcome the image resolution limitations seen with older devices, opening a new field of mobile teledermatology, with two approaches in use. Store-and-forward (SAF) teledermatology allows transmission of images and text to a clinician for review. Live video conferencing (LVC), on the other hand, allows patient and physician to meet virtually at the same time using a webcam or mobile phone camera.

### Determination of Diagnostic Accuracy

Introducing any new diagnostic method in patient care requires testing to ensure the diagnostic accuracy is at least comparable to the accepted standard. Evaluating diagnostic accuracy in dermatology is quite complex. A clinical diagnosis made by a specialist of a lesion as benign is accepted as the reference standard. When a biopsy is performed, however, the reference standard is clearly the histopathology result. Yet even among pathologists, there may be considerable discord in distinguishing between melanoma and benign melanocytic lesions. A review of 392 cases in 2010 in the United States revealed discordant results between pathologists in 14.3% of cases [5]. A 2016 study from another US center indicated discord in 114 of 588 cases (19.4%) [6]. Given the level of disagreement between histopathologists, including those specializing in dermatohistopathology, studies investigating the diagnostic accuracy of a new method, compared with current standards, must be interpreted cautiously [5-7]. Misdiagnosis obviously can have serious impact on patients, but it also complicates studies of newer diagnostic methods.

### Diagnostic Accuracy of Teledermatology

A 2016 review systematically analyzed 21 studies, comparing teledermatology diagnoses using SAF or LVC with results of histopathology or, for nonexcised lesions, clinical diagnoses from face-to-face (FTF) encounters [8]. Overall, FTF diagnosis performed slightly better (67% to 85% agreement with the reference standard, Cohen kappa=0.90) compared with teledermatology (51% to 85% agreement, kappa=0.41–0.63) for the diagnosis of skin cancer. However, several studies have reported teledermatology is more accurate, in some cases even better than in FTF encounters, perhaps because of the improved resolution of mobile phone cameras [9,10].

In the case of skin cancer, timely management is crucial. A review of 3 studies by Finnance et al [8] reported significantly shorter wait times for melanoma patients assessed by SAF mobile phone technology [11,12] compared with conventional procedures. Patients who were referred using teledermatology triage systems tended to receive primary treatment at the first dermatology appointment and required fewer repeat visits [11,12]. We found no data for LVC on this aspect.

### Diagnostic Accuracy With Mobile Phone Dermoscopes

While dermoscopic evaluation is the clinical practice gold standard for FTF visits and has been proven to increase diagnostic accuracy [13], teledermoscopy requires the patient to purchase a dermoscope to use with a mobile phone even though it may not necessarily be superior to teledermatology alone. In a landmark publication in 2011, Krömer et al [14] reported that teledermoscopy had a very high sensitivity and specificity for both malignant melanocytic lesions (sensitivity, 100%; specificity, 97% to 98%; n=6) and malignant nonmelanocytic lesions (sensitivity, 97%; specificity, 92% to 94%, n=58). There was no significant difference between the clinical and dermoscopic diagnoses based on histopathology as the reference standard. The authors reported that, in terms of detailed diagnoses, there were only 16 discordant diagnoses with teledermatology versus 22 with teledermoscopy [14]. A study by Senel et al [15] found that management plans based on teledermatology did not differ significantly from those developed in a FTF encounter, although the accuracy was significantly improved with a mobile phone dermoscope. Further study will help determine what, if any, value is added by teledermoscopy.

### General Skin Conditions

A number of teledermatology studies have focused on any visible skin condition, including a large proportion of nonmalignant lesions that were not biopsied. In these studies, the reference standard was the clinical diagnosis in a FTF visit, so diagnostic concordance conclusions were limited. Overall, however, both original studies and reviews confirm improved teledermatology diagnostic accuracy, particularly because of improved digital image resolution. These investigators conclude that teledermatology now had a diagnostic accuracy comparable to that in a FTF encounter [9,16,17].

## Discussion

### Comparison of Store-and-Forward and Live Video Conferencing

Data on direct comparisons of SAF and LVC in terms of diagnostic accuracy remain scarce [8]. A 2017 study of 214 patients examined video image resolution, assessing several teledermatology formats for concordance with FTF examination as the reference standard. SAF and uncompressed video results were similar and were significantly better than lower resolution compressed video [18]. Uncompressed video may, therefore, close the resolution gap between LVC and SAF methods, although it requires the user to have a faster internet connection.

### Comparison of Requirements for Store-and-Forward and Live Video Conferencing

SAF and LVC have different requirements which, in turn, affect their suitability for different patient subgroups and ultimate benefit in terms of care [16]. SAF has a number of advantages over LVC in terms of equipment and timing (Table 1), such that it may be preferable in more settings.

### Interpretation of Requirements

SAF appears preferable for both the patient and dermatologist in terms of equipment and time requirements. This would particularly be the case in areas where a fast or stable internet connection is unavailable. SAF, therefore, would likely increase the number of patients for whom teledermatology is available. It might also attract more clinicians to engage in it, since it offers more flexibility than either routine care or LVC. Hook et al [19] noted a particular advantage of the anonymity available with SAF teledermatology, as patients with lesions in sensitive areas (eg, from sexually transmitted diseases) may not be willing to identify themselves to a doctor, potentially delaying diagnosis and treatment.

**Table 1:** Advantages of store-and-forward over live video conferencing in teledermatology.

Requirements	Store-and-forward	Live video conferencing
Availability of internet connection	Can be prepared without internet (ie, photos, history) and uploaded or downloaded at any time.	Simultaneous and continuous internet connection is required for both parties.
Speed of internet connection	Internet speed unimportant.	Slow internet speed may lessen diagnostic accuracy.
Appointment	No appointment necessary as evaluation is asynchronous.	Appointment required for synchronous evaluation.
Webcam or mobile phone camera	Useful but not required. Pictures may be on file or taken with any device.	Webcam or mobile phone camera required for both parties for entire session.
Personal identification	Anonymous access possible.	Identification required for face-to-face consultation.

**Table 2:** Features of store-and-forward and live video conferencing for teledermatology.

Features	Store-and-forward	Live video conferencing
Diagnostic accuracy	Higher for store-and-forward compared with that for low-resolution live video conferencing.^a^	Equally high if high-resolution live video conferencing is used.^a^
Physician-patient interaction	Usually low. Physician response may include asking for more information or other images by text.	Physician can directly ask the patient to perform certain tasks or show certain body regions.
Image resolution	Resolution of photographic images is usually higher.	Video images usually have a lower resolution.
3-dimensional view	Not possible for static images.	Live video feed allows clinician to view lesions from various angles.
Webcam or mobile phone camera	Useful but not required, as pictures may be taken with any device.	Webcam or mobile phone camera is required for both parties for entire session.
Free choice of location	Full flexibility.	Bound to locations with a fast internet connection and appropriate equipment.
Free choice of time	Full flexibility.	Bound to scheduled appointment.
Teledermoscopy	Often conducted.^b^	No published literature.
Wait time	Reduced.	No data.
Cost effectiveness	Higher.	Lower.
Security of data transmission	Most commonly transport layer security protocol (end-to-end encryption)	Most commonly transport layer security protocol (end-to-end encryption)

^a^Depends on setting, reference standard, and technology.

^b^Data on increased diagnostic accuracy inconclusive.

### Comparison of Features of Store-and-Forward and Live Video Conferencing

Teledermatology method preferences may depend on the particular features (Table 2) desired by dermatologists and patients.

In regions where many households have no broadband internet connection, such as Germany and Switzerland, SAF is preferable over LVC because of higher diagnostic accuracy [18]. Inner cities offer a better availability of broadband internet connection than rural areas. In regard to an aging population, this aspect should be reinforced as the average age of the population in rural areas is higher which leads to an increased demand for accessible dermatologic care. In addition, SAF offers more time independence for both patient and clinician, letting older people take their time in setting up the technology before sending in their case. Interaction between patient and clinician about SAF images depends on the software used. In some cases, the dermatologist can chat with the patient to ask questions after reviewing the images. The fact that LVC allows synchronous interaction does not appear to increase its diagnostic accuracy over SAF, even with high-resolution LVC [18]. SAF image resolution is higher and can include teledermoscopy. In fact, a systematic review showed SAF did no harm or even improved accuracy in diagnosing skin cancer [20]. Patients at high risk of melanoma are reportedly very accepting of teledermoscopy [21]. While SAF imaging is not currently available in 3D, sensor-in-motion technology that transfers a stored high-definition video along with standard images may become available. Even static images are useful if they are taken from at least two different angles. From a cost effectiveness standpoint, both methods have been shown to reduce costs, but LVC has been found to be more expensive than an SAF approach due to the need for expensive video conference equipment and more consultation time [22]. With regard to patient comfort, 18% of patients reported feeling uncomfortable and 17% reported feeling embarrassed during LVC [23]. However, in some cases SAF patients reported dissatisfaction with the absence of a face-to-face office visit with a dermatologist and when being photographed by another person [24].

### Conclusion

The authors regard SAF as the standard of care for teledermatology. It is well supported by evidence in the literature and available to more patients than LVC (in terms of both location and equipment), offers greater privacy, reduces wait times, improves access to care, and provides both clinicians and patients greater flexibility than traditional clinic visits. Nonetheless, teledermatology is complementary to and not a replacement of FTF clinical encounters.

## Abbreviations

FTF: face-to-face
SAF: store-and-forward
LVC: live video conferencing
